# The Influence of Intrinsic Motivation and Synergistic Extrinsic Motivators on Creativity and Innovation

**DOI:** 10.3389/fpsyg.2019.00137

**Published:** 2019-02-04

**Authors:** Carmen Fischer, Charlotte P. Malycha, Ernestine Schafmann

**Affiliations:** International School of Management, Dortmund, Germany

**Keywords:** creativity, innovation, intrinsic motivation, synergistic extrinsic motivator, relational rewards, transactional rewards, recognition, performance feedback

## Abstract

Despite the vast amount of research focusing on intrinsic and extrinsic motivation, the effects of extrinsic motivators on creativity and innovation have been scarcely investigated. Extrinsic factors can be seen as synergistic extrinsic motivators when they have a positive effect on the outcome. The present study investigates synergistic extrinsic motivators that organizations can use to foster creativity and innovation of their intrinsically motivated knowledge workers. The analysis is based on Amabile and Pratt’s dynamic componential model of creativity and innovation in organizations combined with elements from Ryan and Deci’s self-determination theory. The quantitative data stemmed from 90 knowledge workers of an international consulting company who participated in an online self-assessment. In exploratory factor analyses, extrinsic motivation items consolidated two factors “relational rewards” and “transactional rewards”, while creativity and innovation items resulted in a one-factor solution, called “creativity/innovation performance”.

The results of hierarchical regression analyses confirmed the widely found positive effects of intrinsic motivation on creative and innovative performance. Moreover, the results supported the hypothesis that the extrinsic motivator, relational rewards, moderated the relationship between intrinsic motivation and creativity/innovation performance significantly and positively. The findings showed the higher the perceived probability of receiving relational rewards and the higher the intrinsic motivation, the greater the positive effect on creative/innovative outcomes. At the same time, the results did not confirm the hypothesis, that the moderator transactional rewards had a statistically significant effect on the relationship between intrinsic motivation and creative/innovative performance. Finally, the empirical evidence provided practical implications on how to stimulate the creativity/innovation performance of knowledge workers within organizations.

## Introduction

As work is becoming more and more dynamic and knowledge-based, organizations increasingly depend on creative ideas and innovative impulses from their employees. Knowledge workers’ creativity and innovation are critical for the organizational competitive advantage as they help to enhance a firm’s performance, product quality, and innovative power (Anderson et al., 2014; Liu et al., 2016). Creativity is generally seen as the generation of useful and novel ideas while innovation implies the implementation of these ideas (Anderson et al., 2014).

Research has shown that three factors increase creativity in particular: Motivation, skills, and creativity-relevant processes (Hirst et al., 2009; Richter et al., 2012; Amabile and Pratt, 2016). Generally speaking, motivation is seen as “the heart of organizational behavior” (Gagné, 2014, p. 414) because employees’ motivation has a substantial impact on their performance and productivity (Cerasoli et al., 2014; Amabile and Pratt, 2016). Motivation guides the direction, intensity, and persistence of performance behaviors and can be categorized into intrinsic and extrinsic motivation (Cerasoli et al., 2014; Deci et al., 2017). Extrinsic motivation leads to engagement when material or social considerations are expected (Amabile et al., 1994). Contrarily, when intrinsically motivated, employees perform tasks out of interest and enjoyment for its own sake (Deci et al., 1999; Amabile and Pratt, 2016).

Throughout the last three decades, the positive impact of intrinsic motivation on creativity and innovation was highlighted while extrinsic motivation was often seen as controversial and has been less investigated in this context (Amabile et al., 1995; Anderson et al., 2014). Nevertheless, employers cannot assume that their employees are always intrinsically motivated as relatively few people find their jobs interesting enough to work without getting paid or receiving other rewards in return (Deci et al., 2017). Consequently, in order to enhance creativity and innovation deliberately, extrinsic motivators must also be considered. Contextual factors, like HRM practices, are meant to influence employees’ motivation and thus, to impact outcomes like creative and innovative performance (Byron and Khazanchi, 2012; Cerasoli et al., 2014; Ryan and Deci, 2017). Research evidence on what kind of external motivators foster and impede motivation and furthermore, creative and innovative performance still yields mixed results.

The best-known theory of creativity is Amabile’s model of creativity and innovation in organizations from 1988 (Amabile, 1988; Liu et al., 2016). Based upon recent theoretical developments within the creativity and innovation field the model has been updated by Amabile and Pratt (2016). Complemented with new research findings like synergistic extrinsic motivation and an emphasis on both constructs creativity and innovation, this model represents a promising conceptual framework for the current research scope. According to the concept of synergistic extrinsic motivation, extrinsic motivators can add positively to intrinsic motivation and other outcomes like creativity and innovation (Amabile and Pratt, 2016).

Although Amabile and Pratt (2016) provide a general creativity and innovation framework, they do not elaborate on the different types of motivation and motivators in detail. In order to close this gap, the SDT by Ryan and Deci (2000) can be employed. The SDT distinguishes different motivation types while addressing the link between motivation and performance. Additionally, the theory reflects how multiple factors like pay contingent and managerial styles impact this relation (Deci et al., 2017). So far, no empirical study was found that has already combined Ryan and Deci (2000) and Amabile and Pratt (2016) models in one research scope.

To summarize, the objective of this article is to clarify the open research question about the role of extrinsic motivators on creative and innovative performance as well as their interplay with intrinsic motivation. Extrinsic motivators in the form of specific HRM practices, transactional and relational rewards, are analyzed (Grant and Berry, 2011; Amabile and Pratt, 2016; Deci et al., 2017).

## Theory

### Dynamic Componential Model of Creativity and Innovation in Organizations

**FIGURE 1:**
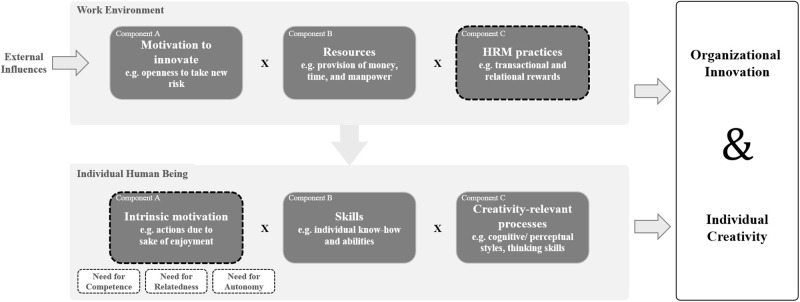
Modified componential model of creativity and innovation in organizations adapted from Amabile and Pratt (2016).

The importance of creativity and innovation is reflected in a multitude of empirical studies, and the number of research efforts has grown significantly over the last 30 years (Amabile and Pratt, 2016; Liu et al., 2016). However, the boundaries between the two concepts of creativity and innovation are still not clearly drawn today (Anderson et al., 2014). Rationales are that focused research and clear, practical guidelines are hampered by the lack of convincing theoretical advances and valid models (Anderson et al., 2014). Amabile and Pratt (2016) recognized this gap and responded by updating Amabile’s well-known model of creativity and innovation in organizations with the latest theoretical developments on motivational factors and their impact on personal and contextual multi-level approaches. New research findings, which are addressed in the 2016 version of the model, include meaningfulness of work, work progress, affect, work orientations, external influences, and synergistic extrinsic motivation (Amabile and Pratt, 2016). It is commonly argued that these factors influence creativity and innovation within organizations (Davis, 2009; Grant and Berry, 2011; Baer, 2012). Their dynamic componential model of creativity and innovation in organizations is a complex, multivariate theory (Amabile and Pratt, 2016). The model (cf. Figure 1 for an adapted version) is broadly clustered into organizational innovation and individual creativity which are displayed as strongly interdependent (Amabile and Pratt, 2016). Both clusters are described with the same three basic multiplicative components that are required to produce something new: Motivation, resources, and processes. The three components of the individual creativity include taking actions due to the sake of enjoyment (intrinsic motivation), individual know-how and abilities (skills), and cognitive/perceptual styles and thinking skills (creativity relevant processes). The three organizational innovativeness components include the openness to take new risks (motivation to innovate), the provision of money, time, and workforce (resources), as well as relational and transactional rewards (HRM practices/processes). Whereas Montag et al. (2012) and Amabile and Pratt (2016) recognize organizational innovativeness and individual creativity as two distinct constructs, others view creativity and innovation as a single construct (Yuan and Woodman, 2010; Soriano de Alencar, 2012).

### Self-Determination Theory

**FIGURE 2:**
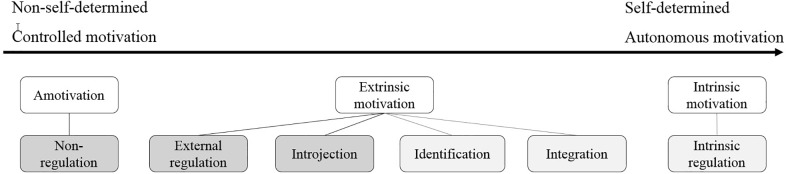
Self-determination theory adapted from Ryan and Deci (2000).

Similar to the theories of creativity and innovation, there is also a variety of motivational theories that partially overlap or contradict each other (Maslow, 1943; Herzberg, 1966; McClelland, 1985; Ryan and Deci, 2000, 2017; Amabile and Pratt, 2016). The theories share the notion that intrinsic and extrinsic motivation are considered as distinct motivational systems. However, depending on the theory, the effects of these motivational subsystems on creativity and innovation as well as on each other are perceived differently. Whereas some researchers like Herzberg (1966) argued that intrinsic motivation (motivators) and extrinsic motivation (hygiene factors) are orthogonal constructs, indicating their independence of each other, authors like Amabile (1993) assume that intrinsic and extrinsic motivation can influence each other and even add up positively. This kind of positive effect is called a synergistic extrinsic motivation effect and is reflected in their latest published model (Amabile and Pratt, 2016). Thus, they argue that extrinsic motivation can also lead to synergistic outcomes. One theory that explains various internal and external motivation types and their dependencies in more detail is the SDT (Ryan and Deci, 2000). The theory suggests that human actions, such as creative and innovative performance, are strongly affected by the type of underlying motivation and are triggered by individual motives and needs. According to the SDT, motivation varies along a continuum between controlled and autonomous motivation(Ryan and Deci, 2000). Autonomous motivation comprises the intrinsic motivation of an employee and the internalized extrinsic motivation. Internalization is defined “as the process of taking in values, beliefs, or behavioral regulations from external sources and transforming them into one’s own” (Ryan and Deci, 2017, p. 182). It is anticipated that internalization of extrinsic motives can also cause similar positive outcomes as intrinsic motivation because it enables self-determination. Ryan and Deci (2000) named these autonomous supporting motivation styles “identification, integration, and intrinsic regulation”. Controlled motivation – on the other side of the continuum – is characterized by non-self-determination which is caused by non-regulation, external regulations, or introjection (Deci et al., 2017). See Figure 2 for visualization of the SDT. Consequently, it is argued that extrinsic motivation is not a one-dimensional construct, as it has often been considered in the past. Thus, previously controversial results of extrinsic motivation effects may have arisen from different views and research settings on extrinsic motivation (Eisenberger and Cameron, 1996; Deci et al., 1999).

The SDT does not only focus on the conceptualization of extrinsic motivation but also on need satisfaction. It consists of six sub-theories that have been tested for decades in numerous work-related studies (Gong and Zhang, 2017; Ryan and Deci, 2017). The BPNT is one of these sub-theories. The BPNT indicates that the autonomous motivation of employees is expected to increase when their basic needs are satisfied in the workplace (Ryan and Deci, 2017). In the case of dissatisfaction of the basic needs, the autonomous motivation decreases and a controlled motivation is anticipated (Ryan and Deci, 2017). It is argued that such controlled motivation has a negative impact on the performance (Ryan and Deci, 2017). Although everybody has needs that trigger motives when salient stimuli are present (Gerrig and Zimbardo, 2016), the level of need satisfaction may vary among individuals. Motives, thereupon, trigger the motivation to act (Gerrig and Zimbardo, 2016). Most need-based theories of motivation postulate very similar basic needs (McClelland, 1985; Ryan and Deci, 2000). The SDT of Ryan and Deci (2000) has built on earlier need theories of Maslow (1943) and McClelland (1985). According to the BPNT, as part of the SDT, there are three basic psychological needs – competence, relatedness, and autonomy – which can be satisfied through self-determination (Ryan and Deci, 2000). The need for competence focuses on the satisfaction of proficiency as well as the feeling of effectiveness in one’s own work (Ryan and Deci, 2002). McClelland (1985) labeled this need the need for achievement. Relatedness provides a feeling of belonging which is supported by cooperation and teamwork (Ryan and Deci, 2002). This need was also mentioned by McClelland (1985), labeled as the need for affiliation. Autonomy represents the choice to engage in an activity that is aligned with one’s values out of personal interest (Ryan and Deci, 2000). Thus, the need for autonomy refers to a need for power over one’s own actions as well as the choice to engage in activities to enable self-fulfillment (Ryan and Deci, 2000). However, the need for power can also be defined differently. McClelland (1985) for instance referred to the need for power as the need to have power over others.

### Intrinsic Motivation and Creative and Innovative Performance

**FIGURE 3:**
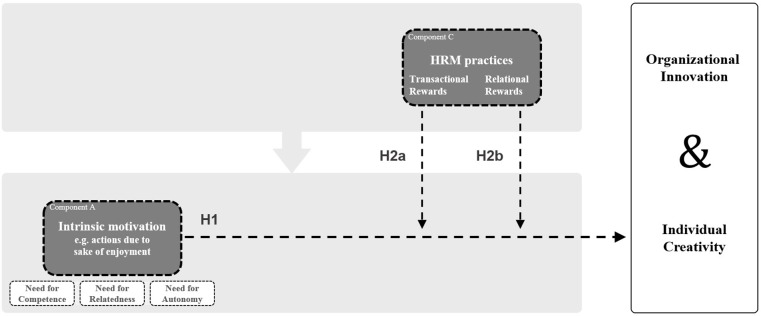
Hypothesized interaction of intrinsic motivation and rewards on creativity and innovation performance.

Intrinsic motivation is characterized by a strong valuation of personal investment and engagement (Ryan and Deci, 2017). Several meta-analyses have shown that the effect between intrinsic motivation and creative performance is significantly positive (De Jesus et al., 2013; Cerasoli et al., 2014; Liu et al., 2016). The dynamic componential model of creativity and innovation in organizations of Amabile and Pratt (2016) also underlines this strong relationship theoretically. Additionally, Grant and Berry (2011) found that this positive effect increases when work involves service to others. This study aims to replicate the widely found positive effects of intrinsic motivation on creative and innovative performance, especially with regard to the group of knowledge workers (see Figure 3).

Hypothesis 1: Intrinsic motivation has a significant positive effect on the creative and innovative performance of knowledge workers.

### Extrinsic Motivators and Creative and Innovative Performance

In earlier times, research on extrinsic motivation often supported a negative impact on intrinsic motivation and performance, commonly referred to as the crowding-out effect (Deci et al., 1999; Kohn, 1999). Such crowding-out effects are becoming less dominant as extrinsic motivators receive more nuanced analyses (Condly et al., 2003; Hammond et al., 2011; Ryan and Deci, 2017). Nevertheless, decades of research have not provided reliable guidelines and a common understanding of the impacts of rewards on motivation as well as creative and innovative performance. Therefore, scholars have called for further investigations (Byron and Khazanchi, 2012; Cerasoli et al., 2014).

HRM practices are a commonly used way to improve motivation in work set-ups. Rewards, a specific HRM practice, are the most common form of extrinsic motivators in the work environment (Cerasoli et al., 2014). In general, they are provided as a consequence of desired behaviors (Rose, 2014). The most common distinction of rewards occurs in transactional and relational rewards (Baer et al., 2003; Gagné and Forest, 2008; Armstrong, 2012; Joshi, 2016). In the following, empirical research findings of the main effects of each reward type on creative and innovative performance are laid out individually before the focus is set on the interaction effects between these rewards and intrinsic motivation on creativity and innovation.

#### Effects of the Extrinsic Motivator Transactional Rewards on Creative and Innovative Performance

Transactional rewards are tangible rewards and refer to any form of financial compensation (e.g., increase in base pay, bonus, monetary awards, and external training with certifications). Regarding transactional rewards, Condly et al. (2003) meta-analysis supported a significant positive main effect between monetary rewards and general performance. Eisenberger and Shanock (2003) found that expected monetary rewards can enhance creativity – a specific form of performance – when participants understand the necessity of performing creative actions, either through instructions or prior experience. These results are consistent with the findings by Deci and Ryan (2014). They found that bonuses for acknowledging the work of individuals are very effective when these knowledge workers expect a bonus. Other researchers, like Malik et al. (2015), found controversial results: Although rewards in general correlated significantly and positively with creativity, financial rewards showed no significant effect on creativity. Malik et al. (2015) explained this finding with the lack of salient transactional stimuli.

#### Effects of the Extrinsic Motivator Relational Rewards on Creative and Innovative Performance

Unlike transactional rewards, relational rewards are intangible. Thus, relational rewards go beyond financial considerations. They include praise, recognition, and performance feedback (Armstrong, 2012), for example in the form of thank-you cards, hall of fame postings, announcements in newsletters (Armstrong, 2012), or funding a successful team for a particular project that the team appreciates, to mention some (Amabile and Pratt, 2016). Such rewards require interpersonal skills and depend on managerial and collegial behavior in order to build valued relationships (Stajkovic and Luthans, 2001; Armstrong, 2012). Therefore, due to the personal component, it is argued that relational rewards are harder to be imitated by competitors than transactional rewards (Armstrong, 2012). Moreover, transactional rewards “only” require the definition and one-time implementation of the specific financial rewards, whereas relational rewards are continuously time-consuming for managers. Thus, from an organizational perspective, it is argued that both types of rewards differ strongly regarding efforts and competitive advantage. The meta-analyses by Hammond et al. (2011) and Byron and Khazanchi (2012) supported that relational rewards in a controlled motivational environment could have no impact or even negative ones on creative and innovative performance. However, in terms of autonomous motivational work set-ups, supportive feedback and the recognition of managers contribute significantly positive to creative outcomes (Madjar et al., 2002; Amabile et al., 2004; Byron and Khazanchi, 2012; Zhang et al., 2017). Evidence for such a positive main effect explicitly for innovation is provided by Taggar (2002).

#### Interaction Effects of Extrinsic Motivators and Intrinsic Motivation on Creative and Innovative Performance

Amabile (1993) stated that the above-mentioned positive boosting effects with extrinsic motivators are more likely when intrinsic motivation is high. In addition to the empirical investigations about the main effects in these contexts, the focus of the present study is therefore on the interaction effects with intrinsic motivation. Cerasoli et al. (2014) showed in their meta-analysis that the significant relationship between intrinsic motivation and general performance was stronger when rewards were granted. However, neither performance nor the type of reward was specified in more detail in their meta-analysis. Amabile and Pratt (2016) assumed a similar interaction effect between intrinsic motivation and extrinsic motivators especially in terms of creative and innovative performance. Therefore, the following is hypothesized (see also Figure 3):

Hypothesis 2a: Transactional rewards moderate the relationship between intrinsically motivated knowledge workers and their creative as well as innovative performance positively.

Hypothesis 2b: Relational rewards moderate the relationship between intrinsically motivated knowledge workers and their creative as well as innovative performance positively.

## Materials and Methods

**FIGURE 4:**
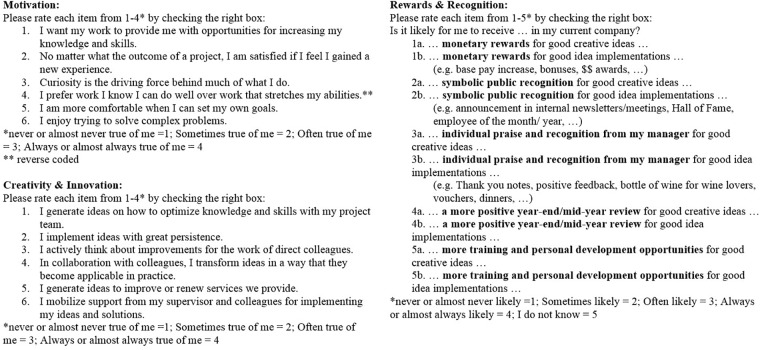
Online self-evaluation questionnaire (Inquery).

The data was collected through an online self-assessment. The English questionnaire (see Figure 4) was sent by e-mail to knowledge workers of a global business consulting firm working in Germany, Austria, and Switzerland. Participants were informed about the purpose of the survey, while anonymity and confidentiality of their data were assured. No incentives for participating in this survey were given. Additionally, the survey instructions emphasized that there were no right or wrong answers to the questions. One hundred and seventy-five consultants received the questionnaire whereby 120 returned it. Thirty of these were excluded because they had either chosen “I just want to look at all the questions” (*N* = 2) or had not answered all questions completely (*N* = 28). Participants who stated “I do not know” for the reward items were excluded listwise. Thus, for the hierarchical regression analyses, only 82 and 87 questionnaires were considered for transactional and relational rewards, respectively. The average age of the participants was 28.27 years (*SD* = 5.62) with an average job tenure in their current organization of 2.20 years (*SD* = 2.05). In the sample 42.2% were women. 95.6% of the participants were graduates. This result represents the intended sample of highly educated knowledge workers. Table 1 provides the sociodemographic characteristics of this sample.

**Table 1:** Sociodemographic characteristics of the polled consultants.

*Sociodemographic characteristics*	*N*	*%*	*M*	*SD*
**Gender**				
Female	38	42.2	2.87	0.52
Male	52	57.8	2.89	0.53
**Age (in years)**				
<25	16	17.8	2.73	0.32
25–29	51	56.7	2.90	0.55
30–34	13	14.4	3.06	0.53
>35	10	11.1	2.89	0.52
**Highest education level**				
Non-graduates	4	4.4	2.83	0.47
Graduates	86	95.6	2.89	0.52
**Job tenure at current company (in years)**				
<2	48	53.3	2.89	0.56
2–3	27	30.0	3.00	0.41
4–5	12	13.3	2.69	0.57
>5	3	3.3	2.50	0.17


In order to control for common method bias due to the self-assessment of a single source, the questionnaire was divided into three sections: Independent, dependent, and moderator variables (Podsakoff et al., 2003). To measure the independent variable “intrinsic motivation”, the WPI by Amabile et al. (1995) was applied. The WPI is a widely used measure to assess (intrinsic and extrinsic) motivation at work (Choi, 2004; Spada and Moneta, 2013). It has acceptable re-test reliabilities of more than 0.60 (Abuhamdeh and Csikszentmihalyi, 2009; Robinson et al., 2014). Its items have been applied in many experiments to better understand motivational behavior for creativity and innovation at work (Prabhu et al., 2008; Chen et al., 2010; Stuhlfaut, 2010). Originally, the WPI consists of 30 items. However, due to the focus on intrinsic motivation within this research (originally 15 WPI items) and to avoid survey fatigue, the number of items was reduced to six intrinsic motivation items. Such WPI item reductions have been previously conducted by other authors such as Robinson et al. (2014) (IM_Robinson_ α = 0.71) and O’Shea (2018) (IM_O′Shea_ α = 0.58). These six items were chosen for their relevance to consultants in their work environment. Opportunities to increase their knowledge and skills (IM item 1: Challenge) as well as to solve complex problems (IM item 6: Challenge) are typical parts of knowledge workers’ business surroundings. Additionally, consultants often prefer to take responsibility early on (Schlossbauer, 2017) which enables them to set goals themselves and work autonomously (IM item 5: Enjoyment). Excluded were items like “[w]hat matters most to me is enjoying what I do”. This item was removed, as consultants generally have to work on all issues the client provides them with, irrespective of whether they enjoy it, or not. This item is argued to be more relevant to self-employed people. Moreover, these six items were selected with the aim to cover a broader field of intrinsic motivation. Therefore, no similar worded items like “I enjoy trying to solve complex problems”/“The more difficult the problem, the more I enjoy trying to solve it” were selected as Robinson et al. (2014) for instance did. The scale reliability of the intrinsic motivation items resulted in a Cronbach’s alpha of 0.54 (Guttman’s α = 0.58). This value represents the alpha after the scale was reduced from six to four items. Although this indicates a reliability index below standard according to Field (2017), this value is not unacceptable. Guttman (1945) stated that alpha values are generally below the actual reliability (Sijtsma, 2009). This indicates that the current intrinsic motivation alpha could be higher than 0.54. In addition to this mathematical inaccuracy of alpha, Kline (1999) supported psychological constructs with reliabilities even below 0.70. He considered them as still realistic and acceptable due to the diversity and complexity of constructs being measured. All items were written in the first person and participants were asked to state the extent to which each item describes them best on a 4-point Likert scale ranging from “never or almost never true of me” (1) to “always or almost always true of me” (4).

The research aimed to evaluate the creative and innovative performance at work. Consequently, for the dependent variable creativity and innovation, the focus was set on on-the-job creativity and innovation that arises during daily work. Due to the lack of consensus about the measurement of creativity and innovation among researchers, there is no commonly used measure for these constructs (Nelson et al., 2014; Fisher, 2015). The questionnaire of Dorenbosch et al. (2005) was applied because they were among the first who measured idea generation and idea implementation without having strong correlations. The items with the highest factor loadings (between 0.674 and 0.842) were selected for the current research. All items were written in the first person and measured on the same 4-point Likert scale as the intrinsic motivation items (see Figure 4). In the current sample, Cronbach’s alpha was 0.63 for the three creativity items, 0.58 for the three innovation items, and 0.79 for the combined creativity and innovation items. Consequently, scale reliability for the combined construct was given (Field, 2017).

For measuring transactional and relational reward, no standard measurement exists (Anderson et al., 2014). Transactional and relational reward items from Gagné and Forest (2008) as well as Armstrong (2012) were selected. A distinction between idea generation and implementation for each reward item was made to enable the differentiation between creativity and innovation. Perceptual measures were used in line with previous research to investigate the effects of rewards on creativity (George and Zhou, 2007; Anderson et al., 2014). The relational rewards were divided into symbolic public recognition, individual praise/recognition from the manager, and performance management as suggested by Armstrong (2012). The transactional rewards were divided into monetary rewards as well as training/personal development investments (Armstrong, 2012). See Figure 4 for details. Participants rated the likelihood of receiving the specific rewards on a 4-point Likert scale ranging from “never or almost never likely” (1) to “always or almost always likely” (4). An additional category gave the participants the option to say “I do not know” (5) to increase validity.

In addition, age, gender, job tenure, and education of the participants were controlled. Other control variables were not defined due to the homogeneous sample of knowledge workers working in the same business consulting company and similar working conditions.

## Results

### Preliminary Analyses

None of the sociodemographic variables (age, gender, job tenure, education) correlated significantly with intrinsic motivation or creativity or innovation (see Table 2). Creativity and innovation correlated moderately and significantly with intrinsic motivation (*r* = 0.37, *p* = 0.000), relational rewards (*r* = 0.34, *p* = 0.001) and transactional rewards (*r* = 0.30*, p* = 0.006). The two measures – creative and innovative performance – showed a significant correlation (*r* = 0.75, *p* = 0.000). Generally, all independent and dependent variables were significantly correlated with each other except for intrinsic motivation with transactional rewards (*r* = 0.14, *p* = 0.202). Univariate variance analyses with sociodemographic control variables demonstrated no significant differences between creative and innovative performance of males (*M* = 2.89, *SD* = 0.53) and females (*M* = 2.87, *SD* = 0.52) in this company. Moreover, no significant difference was found between creative and innovative outcomes and the level of education amongst graduates (*M* = 2.89, *SD* = 0.52) and non-graduates (*M* = 2.83, *SD* = 0.47). Similar findings applied to the different age groups as no significant effect was found. In addition, no significant difference was found between participants who worked 2–3 years in the company (*M* = 3.00, *SD* = 0.41) and those who worked more than 5 years (*M* = 2.50, *SD* = 0.17).

**Table 2:** Means, standard deviations, and intercorrelation among study variables.

	*Measure*	*M*	*SD*	1	2	3	4	5	6	7	8	9	10
1	Gender	0.58	0.50	–									
2	Age	28.27	5.62	0.13	–								
3	Highest education level	0.96	0.21	0.25*	0.09	–							
4	Job tenure at current company	2.20	2.05	–0.06	0.38***	–0.30**	–						
5	Intrinsic motivation	3.25	0.44	–0.14	–0.12	0.03	–0.20	0.54					
6	Creativity	2.87	0.60	0.08	0.06	0.01	–0.13	0.34***	0.63				
7	Innovation	2.90	0.51	–0.05	0.09	0.03	–0.14	0.36***	0.75***	0.58			
8	Creativity/innovation	2.89	0.52	0.02	0.08	0.02	–0.14	0.37***	0.95***	0.93***	0.79		
9	Relational rewards^a^	2.98	0.68	0.05	–0.06	–0.15	–0.06	0.25*	0.36***	0.27*	0.34***	0.86	
10	Transactional rewards^b^	2.11	0.85	0.13	–0.04	–0.03	–0.26*	0.14	0.31**	0.24*	0.30**	0.51***	0.84


*N = 90, ^*a*^*N* = 87, ^*b*^*N* = 82. Cronbach’s alpha is depicted in the diagonal. Coding: Gender: 0 = “female”, 1 = “male”; Highest education level: 0 = “non-graduates”, 1 = “graduates”. Scale from never or almost never true of me/likely (1) to always or almost always true of me/likely (4). ^∗^*p* < 0.05, ^∗∗^*p* < 0.01, ^∗∗∗^*p* < 0.001 (two-tailed).*

The high correlation of 0.75 between creativity and innovation indicated a one-factor solution. This was supported by an EFA. The results showed a Barlett’s Test of Sphericity [chi-square (15) = 148.61, *p* = 0.000] and Kaiser-Meyer-Olkin Measure (KMO) with sampling adequacy of 0.757. This represents a mediocre KMO value, indicating that the variables are suitable for doing an EFA (Backhaus et al., 2016). A principal components analysis with Varimax rotation resulted into a one-factor solution. Overall, this factor explained 49.2% of the variance (eigenvalue = 2.953, Cronbach’s α = 0.79). Therefore, both terminologies were treated as one variable called creativity/innovation performance. This result is in line with Baer (2012) whose findings also showed no significant difference between creativity and innovation. See Table 3 for details.

**Table 3:** Pattern and structure matrix of PCA with varimax rotation for a one-factor solution of creativity and innovation items.

Items	Creativity/innovation performance (*N* = 90)
I: In collaboration with colleagues, I transform ideas in a way that they become applicable in practice.	0.75
C: I generate ideas to improve or renew services we provide.	0.74
C: I actively think about improvements for the work of direct colleagues.	0.72
I: I mobilize support from my supervisor and colleagues for implementing my ideas and solutions.	0.72
C: I generate ideas on how to optimize knowledge and skills with my project team.	0.69
I: I implement ideas with great persistence.	0.59


*C, creativity; I, innovation. Scale from never or almost never likely (1) to always or almost always likely (4). KMO = 0.757. Total variance explained: 49.2%.*

To evaluate the transactional and relational reward items another EFA was conducted. The results indicated a Barlett’s Test of Sphericity [chi-square (45) = 566.94, *p* = 0.000] and Kaiser-Meyer-Olkin Measure (KMO) with sampling adequacy of 0.684. This represents a mediocre KMO value indicating that the variables are suitable for performing an EFA (Backhaus et al., 2016). A principal components analysis with Varimax rotation was done. The EFA was conducted to find a parsimonious solution with a high data fit, meaning to select as little factors with the highest explanation of variance as possible (Tabachnick and Fidell, 2014). Thus, two factors were extracted. Although when following the Kaiser-Kriterium strictly, three factors should have been extracted. This decision was based on three rationales. Firstly, the Kaiser-Kriterium overestimates the number of factors (Field, 2009). Secondly, the third factor had an eigenvalue only slightly above one (eigenvalue = 1.098). Fabrigar et al. (1999) have advised to treat an eigenvalue of one only as a reference point not as a fixed criteria because “it is not really meaningful to claim that a common factor with an eigenvalue of 1.01 is a “major” factor whereas a common factor with an eigenvalue of 0.99 is not” (p. 278). Thirdly, the two-factor solution is in line with the common theoretical distinction between the two constructs transactional and relational rewards (Gagné and Forest, 2008; Armstrong, 2012). The first factor, relational rewards, contained six items, accounting for 34.6% of the variance (eigenvalue = 4.916, Cronbach’s α = 0.86). The factor reflects symbolic public recognition, individual praise from managers, and performance management. The second factor, transactional rewards, accounted for additional 32.8% of the variance (eigenvalue = 1.822, Cronbach’s α = 0.84). It consisted of four items that reflect financial and training investment. Overall, these two factors accounted for 67.4% of the variance. Table 4 provides details about the rotated component matrix of rewards and shows that each creativity (idea generation) and innovation (idea implementation) “item pair” of the reward EFA belongs to the same factor. The high alpha values and factor loadings justified the internal reliability and construct validity (Backhaus et al., 2016).

**Table 4:** Pattern and structure matrix of PCA with varimax rotation for a two-factor solution of reward-items.

Items	Relational rewards (*N* = 87)	Transactional rewards (*N* = 82)
Is it likely for me to receive … in my current company?		
…individual praise and recognition from my manager for good creative ideas…	**0.89**	0.02
…individual praise and recognition from my manager for good idea implementations…	**0.88**	0.02
…symbolic public recognition for good creative ideas…	**0.76**	0.31
…symbolic public recognition for good idea implementations…	**0.74**	0.35
…a more positive year-end/mid-year review for good creative ideas…	**0.56**	0.50
…a more positive year-end/mid-year review for good idea implementations…	**0.56**	0.49
…more training and personal development opportunities for good creative ideas…	0.06	**0.90**
…more training and personal development opportunities for good idea implementations…	0.10	**0.88**
… monetary rewards for good creative ideas …	0.19	**0.70**
… monetary rewards for good idea implementations …	0.29	**0.70**


*Scale from never or almost never likely (1) to always or almost always likely (4). KMO = 0.684. Total variance explained: 67.4%. Primary loadings for each item are in bold.*

### Effects on Creativity/Innovation Performance

Since the sociodemographic control variables were neither significant nor did they influence the outcome of the regression models, they were not considered in further investigations.

The hypotheses were tested within two 3-step hierarchical linear regression analyses on creativity/innovation. In the first regression analysis on creativity and innovation performance, the independent variable intrinsic motivation was entered in the first step, followed by transactional rewards in the second step. Afterward, the interaction between transactional rewards and intrinsic motivation was added (intrinsic motivation × transactional rewards). This model [*F*(3.78) = 8.44, *p* = 0.000] explained a total variance of 24.5% (see Table 5). Intrinsic motivation had a significant effect on creativity/innovation performance (β = 0.38, *p* = 0.000). Intrinsic motivation demonstrated the highest significant beta values of all measures and a strong effect size of *d* = 0.42 (Cohen, 1992). Thus, Hypothesis 1 can be confirmed. Transactional rewards had a significant main effect on creativity/innovation (β = 0.23, *p* = 0.025). However, the interaction effect between intrinsic motivation and transactional reward was not significant (β = 0.17*, p* = 0.089). Thus, Hypothesis 2a cannot be confirmed.

**Table 5:** Hierarchical regression analysis predicting creativity/innovation from intrinsic motivation and transactional rewards.

Predictor	Δ*R*^2^	*R*^2^	*F*	β	*B*	*SE*
Step 1	0.16***	0.16***	14.84***			
Intrinsic motivation				0.38***	0.20	0.05
Step 2	0.06	0.22*	10.91***			
Transactional rewards				0.23*	0.12	0.05
Step 3	0.03	0.25	8.44***			
Intrinsic motivation × transactional rewards				0.17	0.09	0.05


*N = 82. All values are from the last step of the hierarchical regression analysis. ^∗^*p* < 0.05, ^∗∗^*p* < 0.01, ^∗∗∗^*p* < 0.001 (two-tailed).*

**FIGURE 5:**
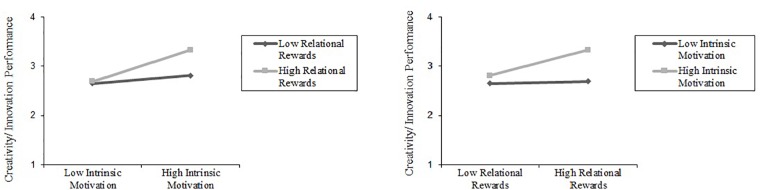
Interaction effects of intrinsic motivation and relational rewards on creativity/innovation performance.

In the second regression analysis on creativity/innovation performance, the independent variable intrinsic motivation was entered in the first step followed by relational rewards in the second step. Then, the interaction of intrinsic motivation with relational rewards was added (intrinsic motivation × relational rewards). This model [*F*(3.83) = 9.70, *p* = 0.000] explained overall 26.0% of the variance. Relational rewards had a significantly positive main effect on creativity/innovation (β = 0.27, *p* = 0.008) with a Cohen’s *d* of 0.52. Relational rewards and intrinsic motivation also had a significantly positive interaction effect on creativity/innovation (β = 0.23*, p* = 0.024). The interaction had an effect size of *d* = 0.59. This represented a medium effect on creativity/innovation performance (Backhaus et al., 2016). Thus, Hypothesis 2b can be confirmed. Figure 5 visualizes this ordinal interaction effect while the exact figures are presented in Table 6.

**Table 6:** Hierarchical regression analysis predicting creativity/innovation from intrinsic motivation and relational rewards.

Predictor	Δ*R^2^*	*R^2^*	*F*	β	*B*	*SE*
Step 1	0.15***	0.15***	14.87***			
Intrinsic motivation				0.38***	0.20	0.05
Step 2	0.06*	0.21*	11.31***			
Relational rewards				0.27**	0.14	0.05
Step 3	0.05*	0.26*	9.70***			
Intrinsic motivation × relational rewards				0.23*	0.12	0.05



*N = 87. All values are from the last step of the hierarchical regression analysis. ^∗^*p* < 0.05, ^∗∗^*p* < 0.01, ^∗∗∗^*p* < 0.001 (two-tailed).*

## Discussion

This study was the first to analyze most common transactional and relational reward items as a moderator of the relationship between intrinsic motivation and the creativity/innovation performance of knowledge workers. The most important finding of this research demonstrates the significant, positive interaction effect of the extrinsic motivator, relational rewards, and intrinsic motivation on creativity/innovation performance. In addition to this significant interaction effect, the main effects between the dependent variable creativity/innovation performance and each of the three independent variables intrinsic motivation, relational, and transactional rewards showed significant positive results.

The results show a strong and highly significant correlation between on-the-job creativity and innovation. This study supports the view that knowledge workers of the international consulting business do not distinguish between idea generation (creativity) and idea implementation (innovation), unlike the two-construct approach of Amabile and Pratt (2016). Apart from the statistical indication, practical circumstances of the consulting business also necessitate that creativity and innovation are handled as a single construct. This business is characterized by consulting services that generally require only a small amount of product design or technical testing. Once generated ideas are put directly into practice, and thus, idea generation and implementation often coincide in time. This finding is not entirely new and complements the existing literature from Yuan and Woodman (2010), who do not strictly distinguish between creativity and innovation. However, the research question remains open as to whether creativity and innovation are considered as one or two constructs in other work environments. The perception of the two terminologies may vary depending on the mental (consulting business) and physical work environments. More research is needed to link the creative and innovative performance of employees with different organizational settings to foster a comprehensive understanding of their interplay (Dorenbosch et al., 2005; Anderson et al., 2014).

### Intrinsic Motivation and Creativity/Innovation Performance

An explicitly strong and significantly positive main effect is found between intrinsic motivation and creative/innovative performance. This implies that the higher the intrinsic motivation, the higher the creative and innovative outcome. This finding confirms the results of earlier research (Hammond et al., 2011; De Jesus et al., 2013; Liu et al., 2016) and supports Amabile and Pratt (2016) model that the individual component “intrinsic motivation” is a critical predictor for creativity. One reason for this significant effect could be that employees who work on perceived inherently interesting tasks enjoy their work, value their personal investment, and dedicate more time to their activities (Ryan and Deci, 2017). Generally, more information is being processed while efforts to develop and implement new and useful ideas are being pursued more persistently (Zhou and Shalley, 2008; Zhang and Bartol, 2010). An additional reason for the significant effect of intrinsic motivation and creativity and innovation performance could be that the work itself involves service to others. Grant and Berry (2011) found that service to others increases the positive effect of intrinsic motivation on creative and innovative outputs. The item “I mobilize support from my supervisor and colleagues for implementing ideas and solutions” could serve as an indicator for supporting the effect stated by Grant and Berry (2011). This item is the only creativity/innovation item that does not explicitly mention service to others. Compared to all other items, this item showed the lowest mean value (*M*_item6_ = 2.76, *SD*_item6_ = 0.75). The highest values are found when improvements for and with the team are targeted (*M*_item4_ = 3.01, *SD*_item4_ = 0.65 and *M*_item1_ = 2.98, *SD*_item1_ = 0.73). Consultants do not only provide service to clients but also help each other on project tasks. Because each project assignment typically has limited resources, success depends on the commitment of each team member. The provision of service to others is promoted by the need for relatedness (Shiraki and Igarashi, 2018). Consequently, it is argued that such a prosocial behavior of consultants satisfies their feeling for relatedness. This, in turn, might increase their intrinsic motivation and so, their creative and innovative outcomes. In addition, Baer et al. (2003), as well as Oldham and Cummings (1996) provided evidence that employees with complex and challenging tasks, such as consultants generally have (Schlossbauer, 2017), show higher intrinsic motivation and thus, greater creative and innovative job performance. By being able to engage in complex and challenging tasks, it is argued that they can prove their competences and abilities which supports their basic need fulfillment. Further research should clarify the assumed role of the different needs in this context.

### Relational Rewards, Intrinsic Motivation, and Creativity/Innovation Performance

The results showed a positive, significant main effect between relational rewards and creative/innovative performance. This result is in line with previous research findings on the relationship between supportive manager feedback/recognition and creative outcomes (Madjar et al., 2002; Gong and Zhang, 2017; Zhang et al., 2017). The following argument can explain the main effect of this extrinsic motivator: Relational rewards initiate salient stimuli strong enough to be recognized by consultants. Without salient stimuli, no creative or innovative action would follow (Gerrig and Zimbardo, 2016). In addition to awareness of the rewards, it is argued that these employees value the relational rewards they receive. Without any appreciation of these HRM practices, less creative and innovative performance would occur (Rose, 2014; Malik et al., 2015). Referring to the dynamic componential model of creativity and innovation in organizations of Amabile and Pratt (2016), the results showed that HRM practices, in the form of relational rewards, have an essential impact on creativity and innovation. Symbolic public recognition, individual praise, and performance feedback are argued to increase a feeling of competence through the evaluation and confirmation of one’s abilities (Ryan and Deci, 2017). It is therefore expected that the satisfaction of the basic psychological need for competence will be met. It is assumed that this increases autonomous motivation and, in turn, leads to better performance (Ryan and Deci, 2017).

In addition to the significant main effect, the results support a significant, medium interaction effect between relational rewards and intrinsic motivation on creativity/innovative performance. The impact of relational rewards on creative and innovative outputs is notably greater when the intrinsic motivation of knowledge workers is high. This finding supports the assumed boosting effect on performance from Amabile (1993). Additionally, no crowding-out effect occurred by using extrinsic motivators as defined by Kohn (1999). Therefore, relational rewards, as a synergistic extrinsic motivator, can add positively to intrinsic motivation as suggested by Amabile and Pratt (2016). Also, Herzberg (1966) orthogonal factor assumption differs from the current research findings which support dependencies between intrinsic motivation and extrinsic motivators. One reason for this significant positive interaction effect might be the perceived appreciation of creativity and innovation in the organization. Perception of an environment is subjective and influenced by what an individual sees, feels, and hears (Atkinson, 1964). Perception might change based on past experiences (Zhou and George, 2001; Dorenbosch et al., 2005). In order to respond to the perceived circumstances, a stimulus – strong enough to trigger motivation – must be present (Gerrig and Zimbardo, 2016). In this context, it is argued that the highly intrinsically motivated knowledge workers perceive that their organization values creativity and innovation. Applying recognition and performance feedback to communicate the appreciation of creative and innovative work is argued to increase employees’ perception and beliefs that creative and innovative efforts are valued within the company (Armstrong, 2012). Therefore, the belief in the importance of creativity and innovation might have influenced employees’ behavior to be more creative and innovative. It is assumed that the likelihood to start new creative and innovative ventures and implement more ideas rises. More attention is given toward making improvements on the job and seeing aspects from different perspectives. This result supports the importance of Amabile and Pratt (2016) organizational component HRM practices.

The theoretical assumption of Amabile and Pratt (2016) on synergistic extrinsic motivators can also be supported with the SDT of Ryan and Deci (2017). When self-determination is given, extrinsic motivators can add positively to the outcome. Self-determination can be reached through the satisfaction of the psychological needs. Several indicators support the need satisfaction of knowledge workers. The highly intrinsically motivated consultants feel most likely satisfied in their need for autonomy due to task ownership and their willingness to take responsibility early on (Schlossbauer, 2017). Additionally, their feeling of competence is triggered by the usage of their know-how and is argued to rise further with verbal praise and feedback because it complements a confirmation of competence. Moreover, it is anticipated that project-oriented employees fulfill their need for relatedness in their project environment, by providing support to their colleagues and clients (Shiraki and Igarashi, 2018). Since the three basic psychological needs have not been empirically tested, it is recommended that future research should specifically analyze their interplay with creative and innovative behavior. Additionally, an emphasis should be set on the different extrinsic motivation types of the SDT from Ryan and Deci (2000). The exact and diversified understanding of work motivation with its subsystems should continue to evolve (Kanfer et al., 2008).

### Transactional Rewards, Intrinsic Motivation, and Creativity/Innovation Performance

The data indicated a significant positive main effect between transactional rewards and creative/innovative performance of knowledge workers. This means the higher the transactional rewards, which implied financial and training investments, the higher the creative and innovative outcome. This result is controversial to Malik et al. (2015) who found no significant main effect when analyzing financial rewards. This finding is aligned with previous research findings by Condly et al. (2003) on the positive, significant relation between monetary rewards and work performance. However, neither Condly et al. (2003) nor Malik et al. (2015) performed a cost-benefit analysis to validate the transactional reward program. A reason for the significant main effect might be that consultants generally expect a bonus as part of their annual salary for a job well done. According to Deci and Ryan (2014), such usage of bonuses to acknowledge individual good work is very effective. However, it is argued that the valuation of bonuses is a pre-requisite for their effectiveness because, without any appreciation of these HRM practices, creative and innovative performance would not be likely to occur (Rose, 2014; Malik et al., 2015). Thus, besides the relational rewards, transactional rewards as a HRM practice can also foster creativity and innovation.

No statistically significant interaction between transactional rewards and intrinsic motivation on creativity/innovation was supported. This indicates that transactional rewards do not have to imply a synergistic nor a crowding-out effect. The first rationale for this non-significant interaction effect might be that there is no formal creativity-/innovation-contingent rewards and recognition within the sampled consulting organization. The findings of Eisenberger and Shanock (2003) provide evidence that monetary rewards only increase creativity when employees are aware of the necessity as to why creative performance should happen. This finding is aligned with Malik et al. (2015), who found that rewards need to be present and perceived as relevant to influence creative and innovative performance significantly. Based on current results, it can be argued that the link between these tangible rewards and the commitment to pursue more creative and innovative work may not be specific and clear enough. A second reason for the non-significant effect could be that the standard deviation of 0.85 is very high within a scale from 1 to 4. This number indicates that employees perceive the likelihood of receiving a reward very different among each other. On average, only about one-third of all employees in a company receive rewards (CEB, 2014). Statistically, the remaining two-thirds of employees consider the likelihood of receiving transactional rewards to be low. Therefore, it is argued that the awareness, salience, and accessibility of the creativity-contingent transactional rewards, combined with strong intrinsic motivation, seem to be too little to cause a significant result.

In summary, the two extrinsic motivator effects support the assumption of Amabile et al. (1995) that “the motivational structure is probably more complex than the simple intrinsic-extrinsic distinction suggested by the literature” (p. 957). The results for relational and transactional rewards are also aligned with the SDT of Ryan and Deci (2000) which distinguishes between different types of extrinsic motivation with various effects. The results show that extrinsic motivators can have a positive effect on intrinsic motivation and creative/innovative performance (relational rewards), however, can also have no effect (transactional rewards).

### Limitations

When interpreting these results, four main limitations have to be considered. First, the research used self-measurements for all variables as the sole and primary data source. Therefore, the reliability of the data may have been compromised. Although self-evaluation is the most commonly used method of analysis at the individual level (Anderson et al., 2014), it might be problematic if employees do not answer honestly. Instead of providing truthful information, they could indicate how they would like their motivation and creative and innovative performance to be perceived (Bryman and Bell, 2015). Manager reports could resolve this limitation. However, managers have only limited insight into their subordinates’ behavior, thoughts, and informal performance contribution (Organ et al., 2006). Since only the individuals themselves know best how to perceive their environment, the self-assessment approach seemed justified, as suggested by Organ et al. (2006). In order to minimize distortion and falsification, the anonymity and confidentiality of employees’ data were ensured. For future studies, it is recommended to test the results of the research with longitudinal study designs and to select multi-level approaches that examine on an individual, team, and organizational level – and thus, enrich the database.

Second, this study might be considered limited in its scale reliability for the motivational sub-systems. Many academics only consider a Cronbach’s alpha value of 0.70 or higher to show a satisfactory internal consistency (Field, 2017). Not all alpha values measured in this study met this criterion. While the constructs creativity/innovation performance, as well as transactional and relational rewards, showed acceptable scale reliability of minimum 0.79, the corresponding value for intrinsic motivation did not fulfill this criterion (Guttman’s α = 0.58). Nevertheless, besides the fact that intrinsic motivation had such high importance for the investigated model that it could not be excluded from the analyses, 0.58 is still argued to be an acceptable reliability because the calculated alpha values are generally below the actual reliability (Guttman, 1945). Moreover, intrinsic motivation presents a psychological construct. According to Kline (1999), such constructs with reliabilities even below 0.70 are still considered as realistic and acceptable due to the diversity and complexity of constructs being measured.

Third, this research has explicitly analyzed intrinsic motivation and extrinsic reward motivators. Extrinsic motivators are directly related to concrete HRM practices, and thus, represent ways in which companies can influence creative and innovative performance. Hence, the focus has been on these constructs. Gerrig and Zimbardo (2016) assume that extrinsic motivators are a prerequisite of extrinsic motivation. Nevertheless, extrinsic motivation was not directly measured. Future research should empirically measure and compare a more sophisticated breakdown of different motivational systems in relation to creative and innovative performance. For example, Ryan and Deci (2000) four different types of extrinsic motivation that fall along a continuum between autonomous and controlled motivation can guide future research.

Fourth, these research results may be limited to the creativity and innovation performance of knowledge workers in a given consulting firm. Generalization issues might occur due to the purposely, non-random sampling of the survey participants as they were generated through the personal business network of one of the researchers. This method was used for reasons of accessibility and resource constraints, as it was the case in several other studies (Choi et al., 2009). For future studies, however, it is recommended to apply different companies and industries. These would enable the analysis of causal inference related to the findings across various industries. Furthermore, future research should shed light on whether different ages of knowledge workers have an impact on their creative and innovative performance.

### Practical Implications

The results supported the positive impact intrinsic motivation has on creativity and innovation. However, because not every employee has an inherently interesting job, employers cannot rely solely on the intrinsic motivation of their employees. In order to promote creativity and innovation in a targeted manner and to make use of this often untapped human potential, extrinsic motivators should also be considered. In particular, leaders are strongly advised to understand the needs of their employees, as well as to be familiar with the organizational targets in order to implement effective HRM practices (Joshi, 2016). Thus, leaders should support the internalization of their employees’ goals with the organizational goals by fulfilling the need for autonomy, competence, and relatedness (Ryan and Deci, 2017). The research findings suggested that HRM practices in the form of individual praise, symbolic public recognition, and performance feedback along with intrinsic motivation foster the creative and innovative outcomes of knowledge workers. Specifically, leaders could enhance their employees’ creative and innovative performance by providing, for instance, constructive feedback or thank-you cards as well as by funding of a successful team in order to demonstrate leaders’ appreciation of their employees’ work. However, it should be noted that each company is characterized by specific values and circumstances with different perceptions and behaviors of its employees (Malik et al., 2015). Country-specific and cultural differences may require local adjustments to some extent in order to achieve the intended outcomes. Most important, the reward tools have to be salient for the individuals in order to let creative and innovation actions occur. Additionally, knowledge workers need to appreciate the incentives offered and need to be aware of how rewards can be achieved. It is recommended that creative people are recognized for their creative and innovative efforts. Such an appreciation should be done even if the activity itself does not lead to an innovation of economic value (Amabile and Pratt, 2016). In addition, it should be noted that providing a relational reward to one employee may be perceived as negative by another employee who does not receive a reward (Joshi, 2016). Establishing an effective reward system requires time and perseverance. Overall, the aim should be to create a “win–win” situation by improving the innovative capacity of the organization in relation to the goals of the employees.

## Conclusion

Academics are still at an early stage of understanding the relevance of environmental factors, their relationship to motivational subsystems, and their impact on creativity and innovation (Soriano de Alencar, 2012; Anderson et al., 2014). This survey attempted to make a contribution to these research areas. Overall, these quantitative, cross-sectional research findings help to reduce the ambiguities regarding the synergistic effects of extrinsic rewards and intrinsic motivation on the creative and innovative performance of knowledge workers. The specific external motivators, relational and transactional rewards, and their effects on the relationship between intrinsic motivation and creative/innovative performance of knowledge workers were tested. By applying the SDT and the dynamic componential model of creativity and innovation in organizations, this research provides three contributions to the contradictory literature on motivation, creativity, and innovation:

First, the results confirm the widely found positive effect of intrinsic motivation on the creative/innovative performance of knowledge workers. This relationship remained significant regardless of whether other variables were added to the model. Second, the findings show that extrinsic motivators in the form of relational as well as transactional rewards can have a significant positive main effect on creative/innovative outcomes. Third, with respect to creative/innovative outputs, extrinsic motivators and intrinsic motivation are not necessarily antagonistic and are best considered simultaneously. Particularly relational rewards were found to add a positive, significant effect to intrinsic motivation on creative/innovative output. Thus, relational rewards in the form of symbolic public recognition, individual praise, and performance management can be synergistic to intrinsic motivation in terms of creativity and innovation. Transactional rewards, however, had no significant effect with intrinsic motivation on creative/innovative performance. This indicates that extrinsic motivators are not *per se* synergistic, nor do they have *per se* crowding-out effects with intrinsic motivation as well as with creative and innovative performance.

It is recommended that organizations create a “win–win” situation by enhancing organizational innovativeness and considering their employees’ needs. As every company is characterized by specific values with different employees’ perception, it is of critical importance that employers carefully analyze the needs of their employees as well as the needs of their business to create an effective reward system. This research has shown that relational rewards in particular help organizations to enhance the creative and innovative performance of their knowledge workers, which in turn strengthens companies’ competitive advantages.

## Data Availability Statement

The dataset is available on request. The raw data supporting the conclusions of this manuscript will be made available by the authors, without undue reservation, to any qualified researcher.

## Ethics Statement

The study was conducted according to the ethical rules of the German Psychological Society (DGP – Deutsche Gesellschaft für Psychology) which is the equivalent to the APA. The main ethical principles of the DGP are: No intervention in the personal rights of the polled consultants, who did not belong to a special vulnerable group, happened. Pain, psychological stress, exhaustion, fear, or other negative effects can be excluded to be caused by this research set-up as the survey instructions emphasized that there is no right or wrong answer. Moreover, no drugs, placebos, or other substances were given to the participants. No covered participant observation and active deceptions took place while complete clarification about the research aim, procedure, and results were granted to the polled consultants. Every participant provided his/her informed consent with the first question of the survey. This question stated whether the participants wanted to fill in the full questionnaire or whether they just liked to look at the questions. Moreover, all data was anonymized. No names or initials, just four generic sociodemographic characteristics (job tenure, age, highest education level, and gender) were interrogated. Confidentiality of the polled consultants’ data was assured all the time. No incentives for participating in this voluntary survey were given. As these ethical DGP principals have been considered, no further ethical committee was consulted.

## Author Contributions

Research design and survey execution were done by CF. The theoretical foundation, data evaluation, and discussion were a common work by CF and CM. CF wrote the first draft of the manuscript. The critical review was provided by CM and ES. CF and CM contributed to manuscript revision. All authors read and approved the submitted version. CM and ES have provided their written consent to the submission of the manuscript in this form. CF has assumed responsibility for keeping CM and ES informed of the progress through the editorial review process, the content of reviews, and any revision made.

## Conflict of Interest Statement

The authors declare that the research was conducted in the absence of any commercial or financial relationships that could be construed as a potential conflict of interest.

## Abbreviations

BPNT: basic psychological need theory
EFA: exploratory factor analysis
HRM: human resource management
IM: Intrinsic Motivation
SDT: self-determination theory
WPI: work preference inventory
